# Cytochrome P450 102A2 Catalyzes Efficient Oxidation of Sodium Dodecyl Sulphate: A Molecular Tool for Remediation

**DOI:** 10.4061/2010/125429

**Published:** 2010-07-01

**Authors:** Irene Axarli, Ariadne Prigipaki, Nikolaos E. Labrou

**Affiliations:** Laboratory of Enzyme Technology, Department of Agricultural Biotechnology, Agricultural University of Athens, Iera Odos 75, 11855 Athens, Greece

## Abstract

Bacterial cytochrome P450s (CYPs) constitute an important family of monooxygenase enzymes that carry out essential roles in the metabolism of endogenous compounds and foreign chemicals. In the present work we report the characterization of CYP102A2 from *B. subtilis* with a focus on its substrate specificity. CYP102A2 is more active in oxidation of sodium dodecyl sulphate (SDS) than any other characterized CYP. The effect of SDS and NADPH concentration on reaction rate showed nonhyperbolic and hyperbolic dependence, respectively. The enzyme was found to exhibit a bell-shaped curve for plots of activity versus pH, over pH values 5.9–8.5. The rate of SDS oxidation reached the maximum value approximately at pH 7.2 and the pH transition observed controlled by two p*K*_a_s in the acidic (p*K*_a_ = 6.7 ± 0.08) and basic (p*K*_a_ = 7.3 ± 0.06) pH range. The results are discussed in relation to the future biotechnology applications of CYPs.

## 1. Introduction

Cytochrome P450 monooxygenases (CYPs) play a key role in primary and secondary metabolic pathways and in drug detoxification. CYPs are heme-thiolate proteins which are widely distributed in animals, plants, and microorganisms [1, 2]. They catalyze the oxidation of nonactivated C–H bonds, often in a regio- and stereoselective manner, according to the equation:

(1) $$\text{RH}+{\text{O}}_{2}\underset{2{\text{e}}^{-}}{\overset{2{\text{H}}^{+}}{\to }}\text{ROH}+{\text{H}}_{2}\text{O}.$$

Among the members of the cytochrome P450 family, the monooxygenase from *Bacillus megaterium* (CYP102A1 or P450 BM3), it is well characterised. This protein is self-sufficient monooxygenase composed of an N-terminal heme monooxygenase linked to the C-terminal diflavin reductase domain on a single polypeptide chain [1, 3–6]. The X-ray structure of the monooxygenase domain of CYP102A1 was first solved in 1993 [5]. Since then, several structures of CYP102A1 mutants with and without bound substrates [6] and one including the FMN binding domain [7] have been solved. More recently, a fragment containing the FAD binding site was crystallized and its structure was resolved by X-ray analysis [8].

The genome sequence of the gram-positive model organism *Bacillus subtilis* 168 encodes eight cytochromes P450 [9]. Of these *B. subtilis* P450s, four have been wellcharacterized at the enzyme level. For example, BioI (CYP107H1) is involved in the early stages of synthesis of the vitamin biotin [10]. CYP152A1 catalyzes hydrogen peroxide dependent hydroxylation of long-chain fatty acids [11]. The two other P450 monooxygenases CYP102A2 (accession number O08394) and CYP102A3 (accession number O08336) within the *Bacillus subtilis *genome that shows high similarity to the *Bacillus megaterium* have been recently identified and characterised [12–14]. 

Because of their broad substrate specificity and catalytic diversity, there is an increasing interest to use P450s in biotechnology [14–21]. The products of these enzymes (e.g., enantiomerically pure hydroxylated or epoxidised fatty acids and their derivatives) might find applications in the preparation of fine chemicals for the synthesis of polymers, flavours, fragrances, for the production of pharmaceuticals, or the optimization of lead compounds and existing drugs. 

In the present work we report the characterization of CYP102A2 from *Bacillus subtilis* with a focus on its ability to catalyze the efficient oxidation of sodium dodecyl sulphate.

## 2. Materials and Methods

### 2.1. Materials

The pCR T7/CT-TOPO TA Expression Kit was purchased from Invitrogen (USA). NADPH (tetrasodium salt, ca. 95 %), sodium dodecyl sulfate (SDS), lauric acid, all other organic substrates and crystalline bovine serum albumin (BSA) (fraction V) were obtained from Sigma-Aldrich Co. (USA).

### 2.2. Methods

#### 2.2.1. Cloning, Expression, and Purification of CYP102A2 from E. coli BL21 (DE3) Cells

*B. subtilis* (ATCC 168) was grown at 30°C in a medium containing 1% peptone, 0.5% yeast extract, and 1% NaCl. After 24 h, cells were pelleted by centrifugation and genomic DNA was isolated according to a standard procedure [22]. PCR was used to amplify the full-length gene CYP102A2 from genomic DNA using the oligo primers synthesised to the 5′ region of the gene from the ATG start codon (5′-ATGAAGGAAACAAGCCCGATTCCT CAGCCG-3′) and to the 3′ end of the gene finishing at the CTA stop codon (5′-TTTAGA TCTCTATATCCCTGCCCAGACATG-  3′). The PCR reaction was carried out in a total volume of 50 *μ*L contained 0.1 *μ*M of each primer, 5 ng template genomic DNA, 200 *μ*M of each dNTP, 5 *μ*L  10 × Pfu buffer, and 2.1 units of Pfu DNA polymerase (Promega). The PCR procedure comprised 33 cycles of 2 minutes at 96°C, 2 minutes at 55°C, and 6 minutes at 72°C. A final extension time at 72°C for 20 minutes was performed after the 33 cycles. The resulting PCR amplicon was TOPO ligated into a T7 expression vector (pCR T7/CT-TOPO). The resulting expression construct (pCYP102A2) was sequenced along both strands and was used to transform competent BL21(DE3) *E. coli* cells. *E. coli* cells, harboring plasmid pCYP102A2, were grown at 37°C in 1 L LB medium containing 100 *μ*g/mL ampicillin. The synthesis of CYP102A2 was induced by the addition of 1 mM IPTG when the absorbance at 600 nm was 0.5–0.6. Four hours after induction, cells (approx. 3 g) were harvested by centrifugation at 8000 r.p.m. and 4°C for 20 min. CYP102A2 was purified by a method similar to that described elsewhere [23]. Protein purity was judged by SDS-PAGE. The enzyme was also cloned as a 6His-tagged protein using the same expression vector (pCR T7/CT-TOPO) and employing as 5′ and 3′ end specific primers the sequences: (5′-ATGAAGGAAACAAGCCCGATTCCTCAGCCG-3′) and (5′-TTTAGATCTCTATATCCCTGCCCAGAC-3′). The 6His-tagged enzyme was purified using NTA-Sepharose column according to the standard protocol. Protein purity was judged by SDS-PAGE. Kinetic comparison (results not shown) of the tagged and untagged enzyme showed that the extra 6His residues on the C-terminus did not interfere with the activity and function of the enzyme.

#### 2.2.2. Assay of Enzyme Activity, Protein and Steady-State Kinetic Analysis

Enzyme activities were measured by determining the rate of NADPH conversion to NADP^+^ and following the decrease of absorbance at 340 nm. The final assay volume of 1 mL contained 0.1 M potassium phosphate buffer, pH 7.2; 0.1 mM NADPH; 1.735 mM SDS and sample containing enzyme activity. One unit of enzyme activity is defined as the amount of enzyme that catalyses the conversion of 1 *μ*mol NADPH to NADP^+^per minute at 37°C. Protein concentration was determined at 25°C by the method of Bradford using bovine serum albumin (fraction V) as standard [24].

Steady-state kinetic measurements were performed at 37°C in 0.1 M potassium phosphate buffer, pH 7.2 by varying the concentration of the substrates (NADPH, SDS). Initial velocities were determined in the presence of 1.735 mM SDS, while the NADPH concentration range was 6.6–100 *μ*M. Michaelis-Menten kinetics was observed under these investigated conditions. The kinetic parameters *k*_cat_ and *K*_*m*_ were calculated by nonlinear regression analysis of experimental steady-state data. Turnover numbers were calculated on the basis of active site per 119 kDa. Kinetic data were analyzed using the computer program GraFit (Erithacus Software Ltd.) [27]. When NADPH was used at a fixed concentration (0.1 mM), the SDS varied in the range of 0.34–1.73 mM. In this case the data are best fitted to the Hill function since the curves are nonhyperbolic (sigmoidal curves). For each experimental velocity curve, the *V*_max_ value, *S*_0.5_ (*S*_0.5_ is the substrate concentration at which *v * = 0.5*V*_max _), and the Hill coefficient, *n*_*H*_, were determined by fitting the plotted *v* *versus* substrate concentration to the Hill equation:

(2) $$v=\frac{V_{\max\;}{\left[s\right]}^{n_{H}}}{{S_{0.5}^{n_{H}}+[s]}^{n_{H}}}.$$

Curve-fits were obtained using the GraFit (Erithacus Software, Ltd.) computer program.

#### 2.2.3. Difference Spectroscopy

Difference spectral titrations were performed in a Perkin-Elmer Lamda16 double beam double monochromator UV-VIS spectrophotometer. Enzyme solution (0.8 mL; 0.077 mg enzyme in 20 mM potassium phosphate, pH 7.4) and enzyme solvent (0.8 mL; 20 mM potassium phosphate, pH 7.4) were placed in the sample and reference black-wall silica cuvettes (10 mm pathlength), respectively, and the baseline difference spectrum was recorded in the range 500–360 nm. Identical volumes of sodium dodecyl sulfate (SDS) solution were added to both cuvettes and the difference spectra were recorded after each addition.

#### 2.2.4. pH Dependence of *V*_max_

The pH profile of CYP102A2 was conducted at 37°C in 0.1 M potassium phosphate buffer (pH 5.9–8.5). p*K*_a_ values were estimated by fitting the experimental data to the equation reported by Blanchard and Cleland 1980 [28], using the computer program GraFit (Erithacus Software Ltd.).

#### 2.2.5. Bioinformatics Analysis and Molecular Modelling

A molecular model of the heme domain of CYP102A2 was constructed using SWISS-MODEL (http://www.expasy.org/swissmod) [29], as described in Axarli et al., 2005 [12]. Atomic contacts in the complex CYP102A2-palmitoleic acid were analyzed by iMolTalk (http://i.moltalk.org/) [30] using a distance threshold set to 4 Angstroms.

## 3. Results and Discussion

### 3.1. Effect of pH on V_max_ for the SDS Oxidation Reaction

P450 monooxygenases catalyze a broad range of reactions, with different members of the family exhibiting quite varied substrate specificity [12, 23]. The enzyme is more active in oxidation of SDS than any other characterized P450 monooxygenase and therefore detailed kinetic analysis was carried out using SDS and NADPH as substrates. 

**Figure 1:**
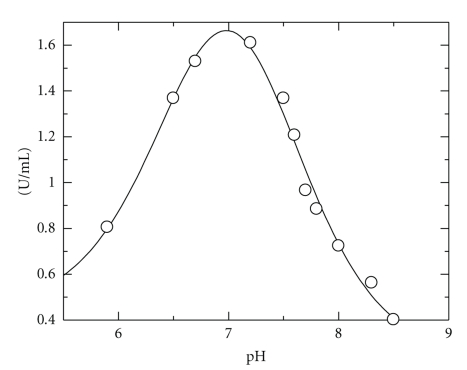
The effect of pH on *V*_max_ of the CYP102A2 SDS/NADPH reaction. Steady-state kinetic measurements were performed in 0.1 M potassium phosphate buffers adjusted to different pH values (5.9–8.5).

The effect of pH on the *V*_max_ for the SDS oxidation reaction was investigated and the results are shown in Figure 1. CYP102A2 remained catalytically active in a broad pH range. The rate of SDS oxidation reached the maximum value approximately at pH 7.2 (0.1 M potassium phosphate buffer). The pH transition observed seems to be controlled by two p*K*_a_s in the acidic (p*K*_a_ = 6.7 ± 0.08) and basic pH range: (p*K*_a_ = 7.3 ± 0.06). The *V*_max _-pH profile yields the p*K*_a_, which reflects the ionization of the enzyme in complex with substrate [29]. As there is no ionizable groups on SDS in the pH range 5.9–8.5, one can conclude that these p*K*_a_s must be for an enzyme side chains.

### 3.2. Effect of SDS and NADPH Concentration on the Enzyme Activity

**Figure 2:**
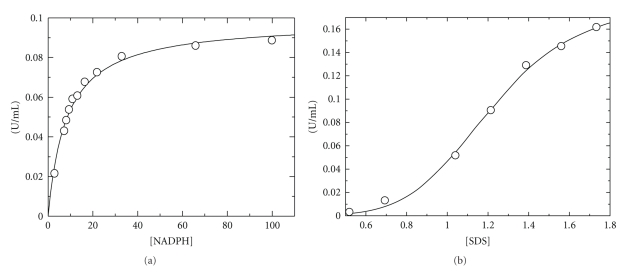
Kinetic analysis of CYP102A2 in 0.1 M potassium phosphate buffer, pH 7.2. (a) Initial velocity analysis with NADPH as variable substrate (6.6–100 *μ*M) and SDS at saturation concentration. (b) Initial velocity analysis with SDS as variable substrate (0.34–1.73 mM) and NADPH at saturation concentration. The plot of rate *versus *SDS concentration is nonhyperbolic and is fitted to the Hill function.

The steady-state kinetics for CYP102A2 was determined by monitoring the SDS dependent oxidation of NADPH. The effect of SDS and NADPH concentration on the reaction rate was studied at 37°C as shown in Figure 2 and the results are listed in Table 1. The dependence of reaction rate on NADPH and SDS concentration was hyperbolic and nonhyperbolic, respectively. Nonhyperbolic data sets showed sigmoidal characteristics and were accurately fitted by the Hill function. Similar nonhyperbolic kinetics was also observed using lauric acid as substrate (Table 1).

 The actual role of nonhyperbolic dependence is not understood. However, it appears more likely that several structural changes throughout the extended hydrophobic active sites in the heme domain of CYP102A2 combine to alter the substrate binding modes in this enzyme and to produce the apparent nonhyperbolic dependence of kinetic on the substrate concentration [5]. Alternatively, the nonhyperbolic dependence probably indicates that CYP102A2 is able to bind simultaneously more molecules of SDS substrates. This which probably shows that the binding process reflects cooperative binding of more than one SDS molecule to the active sites of this enzyme. Atypical (non-Michaelis-Menten) kinetic features have also been observed for several drug metabolising enzyme such as CYP3A4 [5]. In the case of CYP3A4 it has been suggested that this atypical kinetics is due to the existence of, and interaction between, several binding sites on the enzyme. The large CYP3A4 active site may allow the simultaneous presence of multiple molecules and the exact binding conformations appear to depend on the substrates involved, their relative concentration, and affinity for the enzyme. 

**Figure 3:**
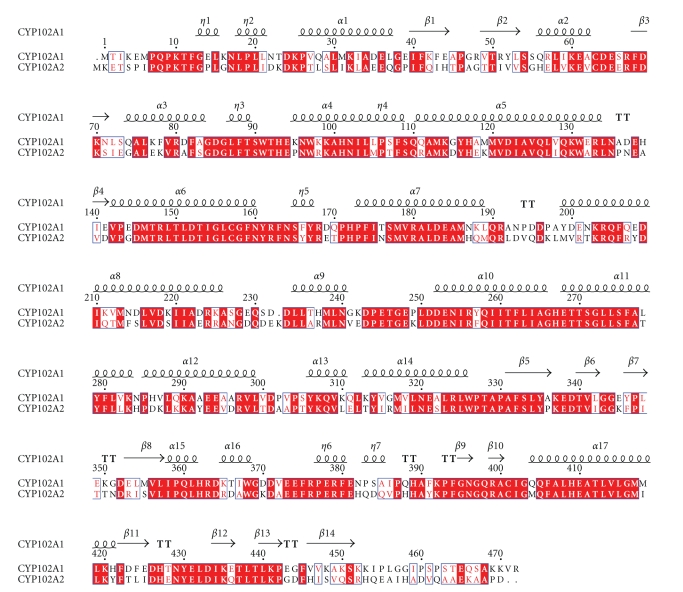
Amino acid sequence alignments. Sequence alignments of heme domain of CYP102A1 (residues 1–472) of *B. megaterium *flavocytochrome P450 BM3 (NCBI accession number A34286) with the respective domain of CYP102A2 from *B. subtilis *(NCBI accession number O08394). The alignments were produced using Clustal W [25] and visualied using ESPript [26]. The secondary structure of CYP102A1 (pdb 1FAG) and numbering are shown above the alignment. Alpha helices and beta strands are represented as helices and arrows, respectively, and beta turns are marked with TT. Conserved areas are shown shaded. A column is framed, if more than 70% of its residues are similar according to physicochemical properties.

CYP102A2 exhibits higher *K*_*m*_ values compared to CYP102A1 towards identical substrates. [23]. Comparing both the amino acid sequences of CYP102A1 and CYP102A2 (Figure 3) and their structures showed significant differences within the substrate access channel, which might explain the different kinetic constants found for CYP102A2. For example, the fatty acid substrate in the crystal structure of CYP102A1 [3] exhibits total 29 interactions. On the other hand the fatty acid substrate in the modeled structure of CYP102A1 exhibits fewer interactions (total 22 interactions). Arg47 and Tyr51 (Figure 3) in CYP102A1 is of particular importance since it is located at the entrance of the active site and interacts with the carboxylate group of the fatty acid substrate [3]. These residues are considered crucial for the proper positioning of the substrate [31, 32]. In CYP102A2, residue 48 (homologous to Arg47 in CYP102A1) is Gly (Figure 3), which rules out a direct effect on substrate binding. In addition, residue 51 (homologous to Tyr51) is Val in our homology model of CYP102A2. Its side-chain is ~6.5 Å away from the substrate which suggests that Val51 might not interact directly with the substrates. Instead of Arg47, Arg353 might interact directly with the carboxylate group of the fatty acid substrate, although the distance between these groups is rather long (~7 Å). 

**Figure 4:**
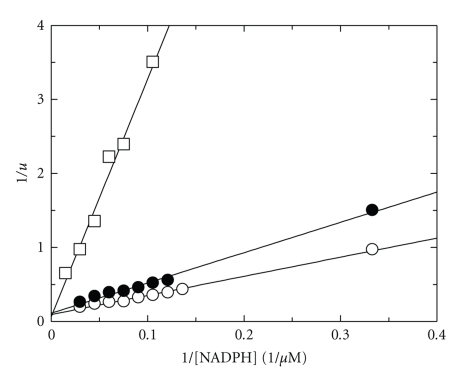
Kinetic analysis of CYP102A2. Initial velocity analysis of CYP102A2 with NADPH as the variable substrate for several fixed concentrations of SDS (mM): 0.7, (○); 1 mM, (•); 2 mM, (□).

CYP102A2 undergoes inhibition by NADPH at concentrations above 0.1 mM. The apparent Michaelis constant is shown in Table 1. When NADPH was the variable substrate with several fixed concentrations of SDS, an intersecting pattern of Lineweaver-Burk plot was obtained (Figure 4). When the slopes and the intercepts of the primary Lineweaver-Burk plots were replotted against the reciprocals of the second fixed substrate, a linear relationship was obtained. Product inhibition studies were conducted with both NADP^+^ and NAD^+^. With NADPH as the variable substrate, NADP^+^ and NAD^+^ gave linear competitive inhibition with *K*_*i*_ equal to 0.1 mM and 149.5 mM, respectively. The intersecting initial velocity pattern obtained and the competitive inhibition obtained using NADP^+^ and NAD^+^ are all consisted with a rapid-equilibrium ordered bi-bi kinetic mechanism.

### 3.3. Spectral Binding Titrations

**Figure 5:**
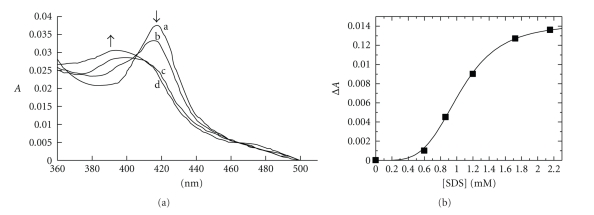
SDS binding to CYP102A2. (a) The spectral changes induced on titration of CYP102A2 with SDS are shown. Arrows indicate directions of change of spectra induced by successive additions of SDS. Difference spectral titrations of CYP102A2 (curve a) and the CYP102A2-SDS 0.8653 mM (curve b) and CYP102A2-SDS 1.726 mM (curve c) and CYP102A2-SDS 2.1553 mM (curve d) complexes. (b) The difference absorbance at 420 nm as a function of the total SDS concentration.

The tight binding of various long chain saturated and unsaturated fatty acids to P450 BM3 has been demonstrated by several groups [13, 23, 31, 32]. Binding of fatty acids to P450 BM3 induces a shift in equilibrium of the heme iron spin state toward the high-spin form, leading to changes in the absorption spectrum in the Soret region [23]. To analyse the binding of SDS to CYP102A2, difference spectral titrations were performed. Figure 5(a) illustrates the spectral perturbations observed for CYP102A2 on titration with SDS. SDS binding showed perturbation of the heme spectrum of CYP102A2, with shift of the resting (substrate free, low-spin) Soret band from 418 nm toward a new position (at 390 nm), typical of the high-spin forms. Gustafsson et al. [23], have reported that comparison of the extent of spin-state perturbation induced between SDS and other fatty acids shows a significant variation. Far more extensive shifts in spin-state equilibrium are observed for the unsaturated and branched-chain fatty acids than with the SDS. 

Plot of the absorption change versus SDS concentration (Figure 5(b)) was fitted accurately to the Hill function, which probably indicates that the binding process reflects cooperative binding of more than one SDS molecule to the active sites of the enzymes. This finding further supports the results observed by kinetic analysis.

In conclusion, in this report we addressed questions regarding the kinetic properties of CYP102A2, a poorly characterized enzyme among the CYP. Detailed studies of bacterial CYPs are justified because of the considerable biotechnological potential of these enzymes. Further investigations of this interesting P450 monooxygenase are now in progress in this laboratory. This includes the isolation and characterization of mutants, obtained by *in vitro* directed evolution.

##  Conflict of Interest

None is declared.

## 4. Abbreviations Used

BMP, the heme domain of P450 BM3; CPR, NADPH: cytochrome P450 reductase; CYP, cytochrome P450; CYP102A1 or CYP102 or P450 BM3, cytochrome P450 monooxygenase from *Bacillus megaterium*; CYP102A2, cytochrome P450 monooxygenase from *Bacillus subtilis*; FAD, flavin adenine dinucleotide; FMN, flavin mononucleotide; NADP^+^, *β*-nicotinamide-adenine dinucleotide phosphate; NADPH, *β*-nicotinamide-adenine dinucleotide phosphate, reduced form; pCYP102A2, the expression vector pCR T7/CT-TOPO which contains the gene of CYP102A2; SDS, sodium dodecyl sulphate.

## Figures and Tables

**Table 1:** Kinetic parameters of CYP102A2 using SDS and NADPH as substrates. Steady-state kinetic measurements were performed at 37°C in 0.1 M potassium phosphate buffer, pH 7.2. All initial velocities were determined in triplicate. The kinetic parameters *K*_cat_ and *K*_*m*_ for NADPH were calculated by nonlinear regression analysis of experimental steady-state data using the GraFit (Erithacus Software Ltd.) program. The *V*_max_ value, *S*_0.5_, and the Hill coefficient, *n*_*H*_, for SDS were determined by fitting the plotted *v* *versus* substrate concentration to the Hill equation using the GraFit (Erithacus Software Ltd.) program.

NADPH		
*k*_cat_ (s^−1^)	*K*_*m*_(*μ*M)	*K*_cat_/*K*_*m*_ (*μ*M^−1^∗s^−1^)
2.05 ± 0.05	8.30 ± 0.62	0.25

SDS		

*S*_0.5_ (mM)	*V*_max_ (*μ*mol/min)	*n*_*H*_
1.22 ± 0.04	0.18 ± 0.0111	5.7 ± 0.66

Lauric acid		

*S*_0.5_ (mM)	*V*_max_ (*μ*mol/min)	*n*_*H*_
0.47 ± 0.005	0.088 ± 0.003	8.7 ± 0.57
